# Metabolomic Study on Nude Mice Models of Gastric Cancer Treated with Modified Si Jun Zi Tang via HILIC UHPLC-Q-TOF/MS Analysis

**DOI:** 10.1155/2019/3817879

**Published:** 2019-06-23

**Authors:** Shanshan Nie, Yuhang Zhao, Xinjian Qiu, Wenbo Wang, Ye Yao, Min Yi, Dongsheng Wang

**Affiliations:** ^1^Institute of Integrated Traditional Chinese and Western Medicine, Xiangya Hospital, Central South University, No. 87 Xiangya Road, Kaifu District, Changsha 410008, China; ^2^Hunan Key Laboratory of Traditional Chinese Medicine for Gan of State Administration, Central South University, Changsha, Hunan 410008, China

## Abstract

Recently, metabolomic methods have been used to explore the complex pathogenesis of cancer and the mechanism of action of traditional Chinese medicine (TCM) formulae. In this study, first, modified Si Jun Zi Tang (MSJZT) was prepared with strict quality control using the instrument method of ultra performance liquid chromatography and photodiode array detector (UPLC-PDA). Subsequently,* in vivo* experiments with tumour-bearing nude mice demonstrated that MSJZT exerted good antitumour effects. MSJZT not only significantly increased mouse body weight but also shrank the tumour volume. Then, the HILIC UHPLC-Q-TOF/MS-based metabolomics approach was used for exploring the pathogenesis of gastric cancer and the molecular mechanism of MSJZT. A total of 59 potential biomarkers in plasma were identified, and 6 pathways were found to be disturbed in gastric cancer. In contrast, after 3 weeks of MSJZT intervention, 32 potential biomarkers were identified, and 4 altered pathways were detected. The changes in glycolytic, amino acid, and lipid metabolisms could be partially regulated by MSJZT through decreasing the content of lactic dehydrogenase (LDH), glutamine synthetase (GS), phosphocholine cytidylyltransferase (PCYT2) mRNA, and protein level. In conclusion, we established a HILIC UHPLC-Q-TOF/MS metabolomic analysis method to demonstrate a complex metabolic profile of gastric cancer. The disordered metabolism could be partially regulated by MSJZT. These findings not only establish a solid foundation for TCM to treat gastric cancer but also provide a basis for further exploration of the precise mechanism of MSJZT activity.

## 1. Introduction

Gastric cancer (GC) is the fourth most common cancer and the second-leading cause of cancer death worldwide [1]. The GC incidence and mortality rate in Eastern countries are the highest, particularly in China, Japan, and South Korea [2]. In 2015, it was estimated that 679,000 new cases and 498,000 new deaths occurred in China. As most patients are asymptomatic, a definite diagnosis occurs at a late stage, and the prognosis is unfavourable [3]. Even with surgery, the 5-year survival of patients is less than 30% [4–6]. Treatments for GC also include chemotherapy and radiotherapy (before and after surgery), which aim to kill cancer cells [7, 8]. However, unfortunately, it is difficult to distinguish cancer cells from normal healthy cells in most cancer treatments, leading to damage of normal cells. Thus, new early screening methods and new therapies for GC are urgently needed.

More than 2000 years ago, practitioners of traditional Chinese medicine (TCM) recorded the clinical manifestations that are now considered GC symptoms, such as anorexia, dyspepsia, weight loss, and abdominal pain [1]. Physicians applied Si Jun Zi Tang (SJZT) to treat gastric disorders, such as chronic gastritis and gastric ulcers, for hundreds of years, because it effectively attenuates nausea, vomiting, and diarrhoea [9]. This famous SJZT formula was first listed in the classical medical book “Tai-Ping-Hui-Min-He-Ji-Ju-Fang” during the Song dynasty. It is composed of four herbal drugs: largehead Atractylodes (*Atractylis macrocephala *Desf.); ginseng (*Panax ginseng* C.A.Mey.); Indian bread (*Poria cocos* (Schw.)Wolf); and Ural licorice (*Glycyrrhiza uralensis* Fisch.). In clinic, SJZT has been used to treat gastrointestinal cancer [10, 11]. Modified Si Jun Zi Tang (MSJZT) is derived from SJZT with the addition of two other herbs, Chinese goldthread (*Coptis chinensis* Franch.) and Oldenlandia diffusa (*Hedyotis diffusa* Willd.), which were added to adapt SJZT for antitumour therapy. Modern pharmacological research has indicated that these herbs have a beneficial effect on cancer therapy [12, 13].

Metabolomics, a significant branch of postgenomic system biology, is used to monitor the endogenous small molecule metabolites of an organism, system, or organ and their association with intrinsic or extrinsic factors [14]. Metabolomics focuses on changes in the overall terminal products, which is consistent with the overall concept of TCM [15–17]. In recent years, it has become a promising technology in the study of human cancers and has been successfully applied in various cancers, such as stomach, colorectal, and pancreatic cancer [18–20].

Therefore, in this study, a hydrophilic interaction liquid chromatography and liquid chromatography quadrupole time-of-flight mass spectrometry (HILIC UHPLC-Q-TOF/MS)-based metabolomics approach was used as an exploration analysis platform to capture the altered endogenous metabolites in the plasma of GC tumour-bearing nude mice and illuminate the antitumour effects and mechanisms of MSJZT.

## 2. Materials and Methods

### 2.1. Preparation of the MSJZT Decoction

All crude herbs were purchased from pharmacy of Traditional Medicine, Xiangya Hospital, Central South University (Hunan, China). The herbs were authenticated by pharmacists Rong Zeng and Wenlong Chen (Xiangya Hospital, Central South University). The detailed information regarding the herbs is provided in Table 1. According to the traditional decocting method, the mixed herbs were soaked in double distilled water for 1 h at a ratio of 1:8 (m/v), then filtered through eight-layer absorbent gauze. The residue was dispersed again in distilled water at a ratio of 1:6 (m/v) and then boiled for 30 min. Finally, the filtrate concentration was adjusted to 1 g/ml by heating evaporation and filtered through a 0.45-micron filter and stored at 4°C.

### 2.2. Chemicals and Reagents

5-Fluorouracil (5-Fu) was purchased from Shanghai Xudong Haipu Pharmaceuticals, China. RPMI 1640 medium, trypsin enzyme, and penicillin-streptomycin solution were purchased from HyClone (Utah, USA). Foetal bovine serum (FBS) was purchased from Sijiqing (Hangzhou, China). Isoflurane was purchased from Rivard Life Technology Co., Ltd. (Shenzhen, China). HPLC-grade methanol and acetonitrile were obtained from Merck (Darmstadt, Germany). Phosphoric acid was purchased from Damao Co., Ltd. (TianJin, China). HPLC-grade water was purchased from Wahaha Co., Ltd. (Hangzhou, China). Ginsenoside Re, pachymic acid, glycyrrhizic acid, atractylenolide III, berberine, and oleanolic acid were all purchased from Solarbio Co., Ltd. (Beijing, China).

### 2.3. Determination of the Content of MSJZT

The levels of the main active ingredients of MSJZT were detected using a UPLC-PDA method. Chromatographic separation was performed on a Waters ACQUITY UPLC system with a photodiode array detector (PDA) (Waters, Milford, MA, USA) and Waters BEH (R) C18 (100 mm×2.1 mm, 1.7 *μ*m) column. The column temperature was set at 30°C. The mobile phase was composed of 0.1% phosphoric acid (A) and acetonitrile (B). The optimum adsorption and elution conditions were set as follows: 0–1.5 min, 95% B; 1.5–6 min, 95–80% B; 7–10 min, 80–60% B; 10–10.5 min, 50–35% B; 10.5–12 min, 35–30% B; 12–12.5 min, 30–20% B; 12.5–16 min, 20–10% B; 16–16.5 min, 10–0% B; and 16.5–18 min, 0–95% B. The corresponding chromatograms are shown in Supplementary Materials.

### 2.4. Cell Line and Cell Culture

SGC-7901, a cell line of moderately differentiated human gastric adenocarcinoma, was kindly provided by Dr. Wei Xia (College of Pharmacy, Central South University). The cells were cultured in an incubator (5% CO_2_, 37°C). The culture medium was prepared with 90% RPMI 1640 medium, 10% FBS, and 1% antibiotics.

### 2.5. Animals

BALB/c-nu/nu male mice (n=43 weighing 16–18 g) were purchased from the Department of Laboratory Animals of Central South University. Mice were fed under specific pathogen-free conditions that strictly followed these conditions: temperature, 22–26°C; humidity, 40–60%; and a 12-h dark-light cycle. All animals were free to consume a normal diet and drinking water* ad libitum*. The protocol was approved by the Medical Ethics Committee, Central South University (Changsha, China).

#### 2.5.1. Xenograft Model of GC

After a week of acclimation, five mice were subcutaneously injected with 5×10^6^ SGC-7901 cells suspension into the axilla of the right forelimb. When the tumour volume reached 100 mm^3^, the tumours were transplanted into another five mice using a puncture needle biopsy. After propagation for 3 generations in mice, the tumours were removed, minced into 1mm^3^ cubes, and placed in precooled RPMI 1640 medium. Seven mice were selected randomly as normal group. The other twenty-one mice were inoculated under a state of anaesthesia using isoflurane inhalation. When the tumour volume reached approximately 100 mm^3^, the mice were randomly divided into 3 groups (n=7 per group): the MSJZT (0.494 g/kg/day) group; the model group; and the 5-Fu (20 mg/kg/day) group. The groups were treated with the corresponding dose of drugs, while the model group was administered equal volumes of sodium chloride injection for 3 weeks. MSJZT and sodium chloride were administered by gavage, while 5-Fu was given by intraperitoneal injection. The tumour size was measured every three days using a digital Vernier calliper. The tumour volume (TV) was calculated by the following formula:

(1) $$\begin{array}{c} TV=a\times b\times \frac{b}{2} \end{array}$$

where a is the length and b is the width of the tumour. After 3 weeks of intervention, the blood and tumour samples were collected and stored at −80°C.

#### 2.5.2. Plasma Preparation for LC-MS

The plasma sample was prepared as follows: first, all samples were thawed slowly at 4°C and vortex mixed with 1 ml of precooled methanol/acetonitrile solution (1:1, v/v) and then subjected to ultrasound for 30 min. Next, the solution was incubated at −20°C for 10 min and centrifuged at 14000 rpm for 20 min. The samples were vacuum dried and redispersed into 100 *μ*l of acetonitrile solution (acetonitrile: water=1:1, v/v). After dissolving, vortexing, and centrifuging, the supernatant was collected for metabolomic analysis.

#### 2.5.3. HILIC UHPLC-Q-TOF/MS Conditions

Metabolomic analysis was performed using an UHPLC (1290 Infinity LC, Agilent Technologies) coupled to a quadrupole time-of-flight (ABSCIEX Triple TOF 5600). For HILIC separation, samples were analyzed using a 2.1 mm×100 mm ACQUIY UPLC BEH 1.7 *μ*m column (Waters, Ireland). In both ESI-positive and -negative modes, the mobile phase contained A (25 mM ammonium acetate and 25 mM ammonium hydroxide in water) and B (acetonitrile). The gradient elution was as follows: 0–1 min, 95% B; 1–14 min, 95%–65% B; 14–16 min, 65%–40% B; 16–18 min, 40% B; 18–18.1 min, 40%–95% B; and 18.1–23 min, 95% B. The ESI source conditions were set as follows: Ion Source Gas 1 (Gas 1) as 60, Ion Source Gas 2 (Gas 2) as 60, curtain gas (CUR) as 30, source temperature: 600°C, and Ion Spray Voltage Floating (ISVF)±5500 V. In the MS only acquisition, the instrument was set to acquire over the m/z range 60–1000 Da, and the accumulation time for the TOF/MS scan was set at 0.20 s/spectra. In auto MS/MS acquisition, the instrument was set to acquire over the m/z range 25–1000 Da, and the accumulation time for the product ion scan was set at 0.05 s/spectra. The product ion scan was acquired using information dependent acquisition (IDA) with high sensitivity mode selected. The collision energy (CE) was fixed at 35 V with ±15 eV. Declustering potential (DP) was set as ±60 V.

### 2.6. Data Preprocessing

First, the raw MS data (wiff. scan files) were converted into mzXML files using Proteo Wizard MS convert and then processed using XCMS for alignment, feature detection, and retention time correction. The metabolites were identified by accuracy mass (<25 ppm), and the MS/MS data were kept for further analysis. In the extracted ion features, only the variables that were more than 50% of the nonzero measurement values in at least one group were kept. For the multivariate statistical analysis, the SIMCA-P14.1 (Umea, Sweden) software was used. After the Pareto scaling, the unsupervised principal component analysis (PCA) and supervised orthogonal partial least-squares-discriminant analysis (OPLS-DA) were performed. The 7-fold cross-validation and response 200 permutation testing were used to evaluate the robustness of the model. The significant different metabolites were determined based on the combination of variable influence on projection (VIP >1) and a significance threshold of two-tailed Student's t-test (*P*<0.05).

### 2.7. Pathway Analysis

Metabo-Analyst 3.0 (http://www.metaboanalyst.ca/) was used to detect the disturbed pathways related to GC. All compound names were matched to the HMDB IDs for the subsequent KEGG pathway analysis and selected for the hypergeometric test and relative betweenness centrality for pathway enrichment analysis and pathway topological analysis. Significant metabolites were hierarchically clustered (average linkage) by Pearson correlation in heatmaps, which were generated by Multi Experiment Viewer Version 4.9.0 (http://www.tm4.org/mev/) [21].

### 2.8. Quantitative Real-Time Polymerase Chain Reaction (qRT-PCR)

According to the manufacturer's instructions, total RNA was isolated from the tumour tissues with Tripure reagent (Aidlab Biotechnologies Co., Ltd., Beijing, China). 5 *μ*g total RNA was used for cDNA synthesis by the RevertAid First Strand cDNA Synthesis Kit (Thermo Scientific, USA). Quantitative real-time PCR with MonAmpTM SYBR® Green qPCR Mix (Monad Biotech Co., Ltd., China) was performed using a CFX Connect system (Bio-Rad, USA). Specific primers are displayed in Table 2. GAPDH was the reference gene and the normalization of data was used the quantitation-comparative 2-ΔΔCT algorithm.

### 2.9. Western Blotting

The tumour tissues were lysed in RIPA buffer (Beyotime Institute of Biotechnology, China) containing Halt™ Protease and Phosphatase Inhibitor Single-Use Cocktail (Thermo Scientific, USA). A BCA assay kit was used to quantify the protein concentrations according to the manufacturer's instructions (Beyotime Institute of Biotechnology, China). Equal protein amounts were electrophoresed on SDS-PAGE gels (Bio-Rad, USA), transferred to a polyvinylidene fluoride (PVDF) membrane (0.22 *μ*m, Biosharp, China), blocked with 5% nonfat milk for 2 h and incubated with lactic dehydrogenase (LDH) (1:2000), glutamine synthetase (GS) (1:1000), phosphocholine cytidylyltransferase (PCYT2) (1:500) (Proteintech, Wuhan, China), and GAPDH (1:10000) (Abbkine, Scientific Co., Ltd., Wuhan, China) for overnight. After incubation with the corresponding secondary antibody for 2 h, the band was subjected to visualize using a ChemiDoc™ MP Imaging system (Bio-Rad, USA) and the results were analyzed by Image Lab software (Bio-Rad, USA).

### 2.10. Statistical Analysis

All data are depicted as the mean ± standard deviation (SD). The measurement data were detected by one-way analysis of variance. Student's t-tests were performed, and P-values were generated for all metabolites.* P*<0.05 was defined as significant. SPSS 22.0 and GraphPad Prism 7.0 software were applied for statistical analysis.

## 3. Results

### 3.1. Quality Control of MSJZT

The supplementary information presents the results of UPLC-PDA analysis of MSJZT. The representative chromatograms are shown in Figure S1. The chromatograms of six reference standards are provided in Figure S2. Tables S1 and S2 show the regression equation of 6 reference standards and the contents of the 6 herbs in MSJZT (mg/g), respectively. These results indicate that the quality of MSJZT was controlled, and the extraction method was stable and reliable.

### 3.2. Body Weight and Food Intake

**Figure 1:**
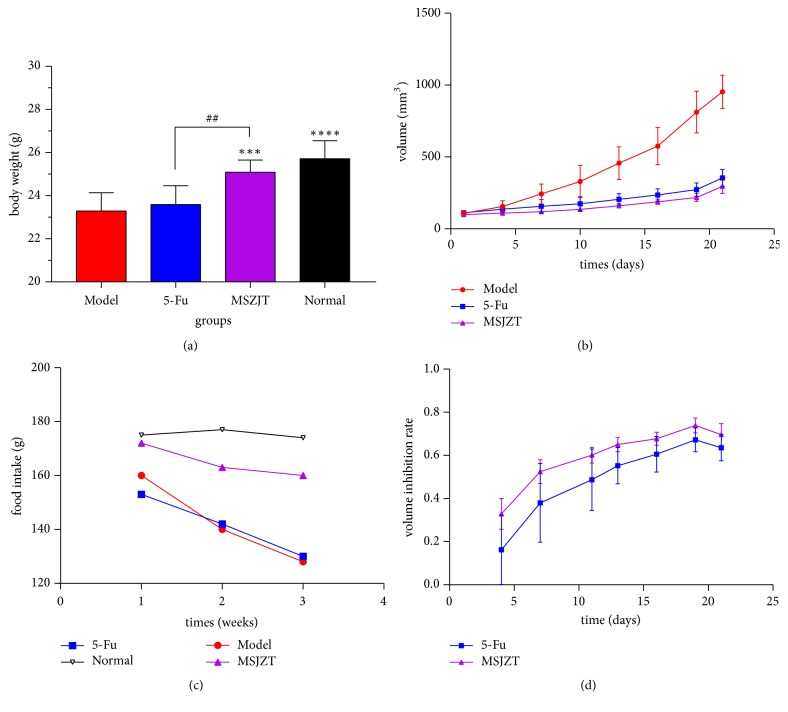
Antitumour effect of MSJZT. (a) The body weight of mice in each group after 3 weeks (n=7). Compared with the model group, *∗∗∗*P<0.01 and *∗∗∗* *∗*P<0.001; ##P<0.01 compared with the MSJZT group; (b) the tumour volume growth curve of mice in the model, 5-Fu, and MSJZT groups. (c) The food intake of the mice in each group for 3 weeks; (d) the volume inhibition rate curves in the MSJZT and 5-Fu groups.

There was no obvious difference in body weight among the groups before treatment (*P*>0.05). As shown in Figures 1(a) and 1(c), after 3 weeks of treatment, a significant difference was detected between the model group and the normal group. Compared with the normal group, the body weight in the MSJZT group was lower, but the difference was not significant. However, the body weight of mice in the 5-Fu group was significantly lower than in the MSJZT group (*P*<0.01); a significant difference was also found between the model and MSJZT groups (*P*<0.01). The quantity of food intake in the MSJZT group was significantly increased (*P*<0.05) compared with the 5-Fu group. This result demonstrated that the tumour-bearing mice were eating less food than normal mice, and MSJZT improved the appetite of the mice.

### 3.3. Tumour Volume Curve

Tumour volumes were measured every three days, and a remarkable disparity existed between the treatment and model groups. As shown in Figures 1(b) and 1(d), the tumour volumes of the treated groups increased more slowly than that of the model group from the fourth day. After 3 weeks of intervention, the tumour growth inhibition rate in the MSJZT and 5-Fu groups reached 68.98% and 62.76%, respectively. The tumour growth inhibition rate was calculated as (1−mean volume of treatment group/mean volume of model group×100%). There was no significant difference between the 5-Fu and MSJZT groups (*P*=0.31). These results indicate that MSJZT has a good antitumour effect.

### 3.4. Histomorphology of the Tumour Tissue

**Figure 2:**
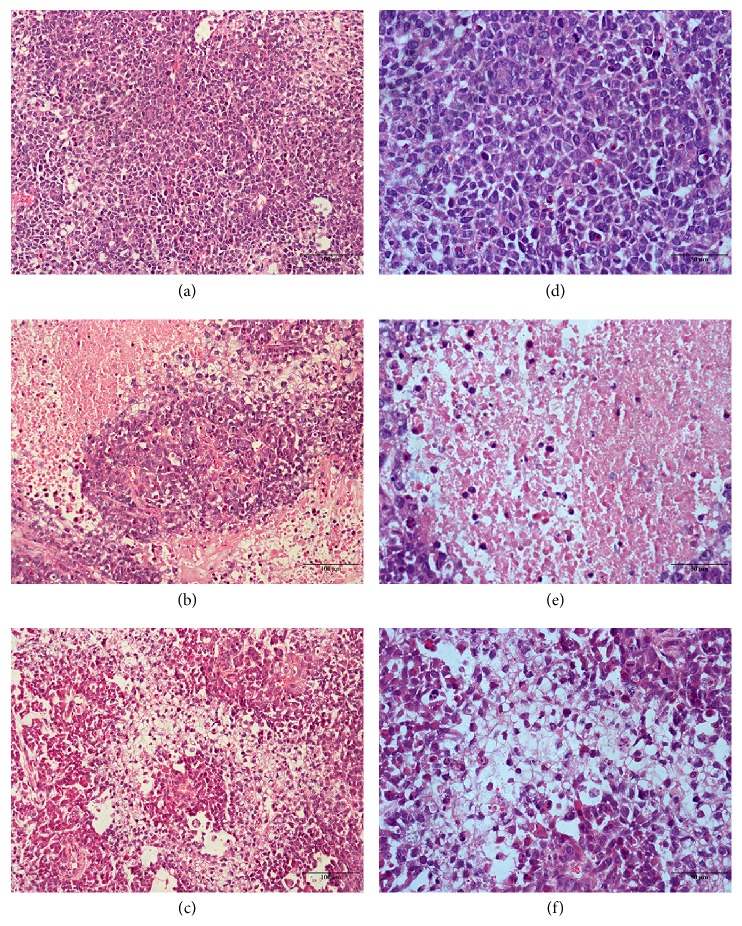
Representative histopathological images with HE method. Model group (a, d), 5-Fu group (b, e), and MSJZT group (c, f). Original magnification ×200 (a–c); ×400 (d–f).

Tumour cell morphology was characterized by both nuclear and cytoplasmic alterations [22]. Figures 2(a) and 2(d) display the morphology of the cancer cells in the model group. These cancer cells had many dark mitotic figures and rows of infiltrating neoplastic cells, with marked pleomorphism and many blood vessels. In the 5-Fu group, the cancer cells had reduced growth activity with large focal necrosis. In the MSJZT group, the cancer cells still had some viability, but the cells were in a state of loose arrangement, and some of the cancer cells were pyknotic, karyolytic, and dead.

### 3.5. Metabolomic Profiling of the Samples

**Figure 3:**
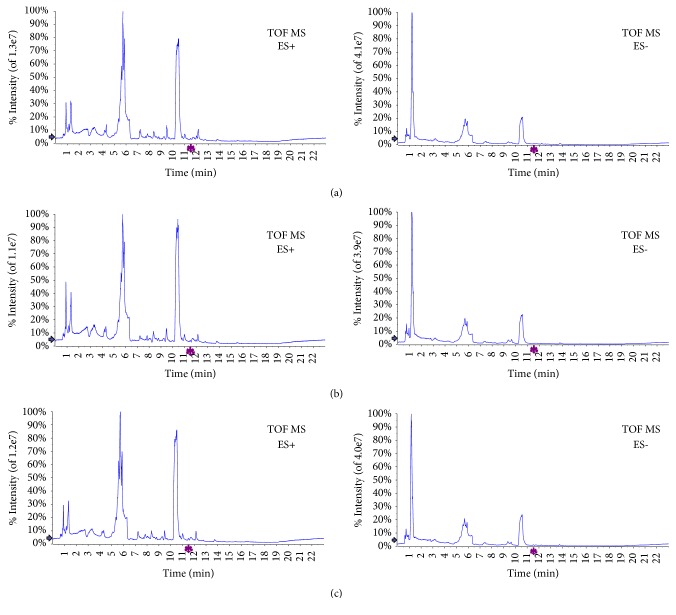
Representative HILIC UHPLC-Q-TOF/MS total ion intensity chromatograms (TIC) of mice plasma obtained from the model group (a), normal group (b), and MSJZT group (c) in both positive and negative ion modes.

**Figure 4:**
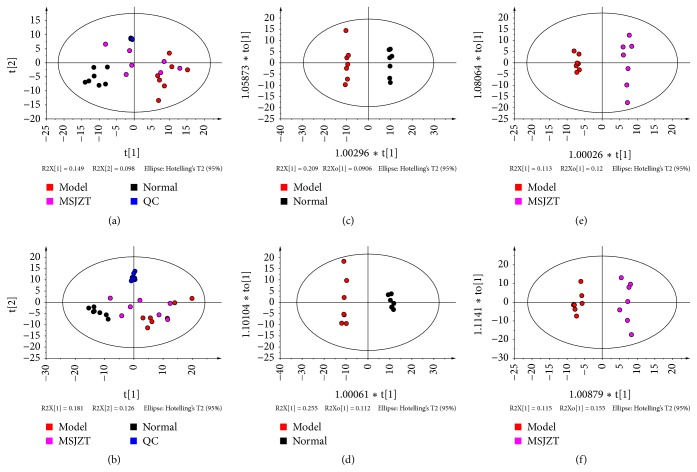
PCA and OPLS-DA score plots. (a, b) The total PCA score plots in both positive and negative ion modes, respectively. (c, d) The OPLS-DA score plots between the model and normal groups in both positive and negative ion modes. (e, f) The OPLS-DA score plots between the model and MSJZT groups in both positive and negative ion modes.

**Figure 5:**
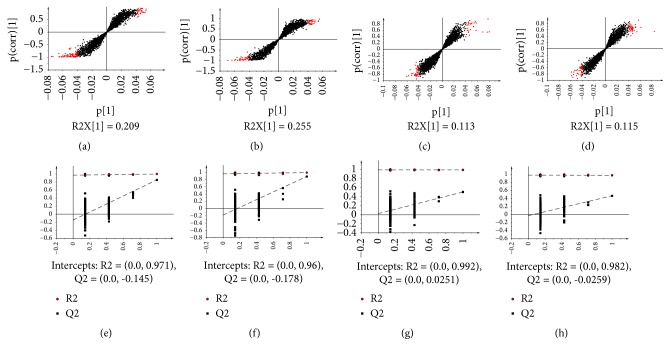
The S-plot of OPLS-DA and 7-fold cross-validation with 200 iterations permutation test. (a, b) represent the S-plot between the model and normal groups in the positive and negative ion modes, (c, d) represent the S-plot between the model and MSJZT groups in positive and negative ion modes, respectively. (a)–(d) The red solid dots represent the potential biomarkers among groups. (e, f) The permutation test of the normal* vs.* the model group in both positive and negative ion modes. (g, h) The model* vs.* the MSJZT group in both positive and negative ion modes.

To further elucidate the antitumour mechanism of MSJZT, we chose the MSJZT, model, and normal groups to conduct metabolomic analysis. Eight replicates of the QC samples were used to evaluate the repeatability of the metabolomic method.  Figure 3 presents the representative total ion chromatograms (TIC) in both positive and negative ion modes. The samples were scattered into different areas in Figure 4, indicating enormous altered endogenous metabolites among the groups. Considering the overlaps between the MSJZT and model groups, OPLS-DA, a supervised pattern recognition method that can maximize the separation among the groups and minimize individual differences, was further employed to determine the changed endogenous metabolites among the model, normal and MSJZT groups. As shown in Figure 4, we found the points of 3 groups were separated from each other both in positive and negative ion modes, and the QC samples were tightly held together. Furthermore, the 200 iterations permutation test shows the good robustness and predictability of the OPLS-DA models in Figures 5(e)–5(h). As the permuted values to the left were lower than the original points to the right, and the Q^2^ intercept was below zero, the OPLS-DA model was verified against overfitting and with good quality [23].  Table 3 indicates the practical model parameters. According to the OPLS-DA model, variables that were satisfied with a threshold of VIP >1 were reserved. Subsequently, the S-plot was established to make the variables visible (Figures 5(a)–5(d)), and every point represented a variable of a three-dimensional dataset. Points situated far away from the origin displayed higher P-values and were regarded as the potential discriminant variables. For each S-plot between two groups, the lower left quadrant represented the higher levels of metabolites in the left group related to the right group; correspondingly, those located in the higher right quadrant displayed the higher levels of metabolites in this group [14, 24]. As shown in Figures 5(a)–5(d), there were some metabolic changes among the groups.

### 3.6. Metabolites Identification

To select the potential biomarkers among the thousands of variables, the criteria must satisfy VIP >1 and the P-value <0.05. Metabolite structural identification was performed by exact matching of mass number (<25 ppm) and second-stage spectra with the laboratory's self-built database. Here, we used glycerol 3-phosphate, for instance, to interpret the identification process. First, we manually removed the redundant substances and isomers. Under the positive ion mode, the potential metabolite was found to be M173T836 according to the retention time of 835.814s and the mass-charge ratio of the primary parent ion m/z, 173.02025. The second-stage spectra of glycerol 3-phosphate were obtained by second-order fragment ion mirror matching. Then, the metabolite was identified as glycerol 3-phosphate, and the matching degree was high, with a score reaching 0.999. In this way, other potential metabolites were identified. Finally, 59 metabolites were putatively annotated as the significant biomarkers to contribute to the difference between the model and normal groups, and 32 metabolites were identified as differential endogenous metabolites between MSJZT and the model groups both in positive and negative ion modes. Tables 4 and 5 show the detailed RT, m/z, VIP, and fold change of identified potential biomarkers among groups in both positive and negative ion modes. As shown in the chart, 30 identified biomarkers were upregulated, and 29 biomarkers were downregulated under GC, while 20 metabolites were upregulated, and 12 biomarkers were downregulated after 3 weeks of treatment.

### 3.7. Metabolic Pathway and Function Analysis

**Figure 6:**
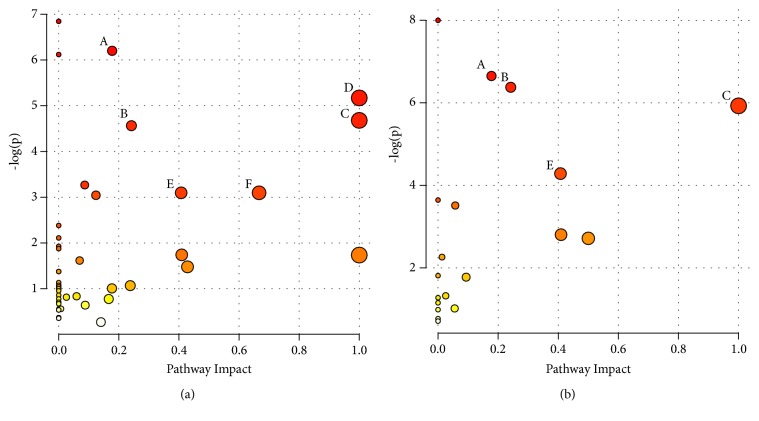
Pathway topology analysis related to GC. (a) The summary of the altered metabolism pathways in the model group. (b) The summary of the altered metabolism pathways in the MSJZT group. Disturbed metabolism pathways include glycerophospholipid metabolism (A), histidine metabolism (B), D-glutamine and D-glutamate metabolism (C), phenylalanine, tyrosine and tryptophan biosynthesis (D), phenylalanine metabolism (E), and valine, leucine, and isoleucine biosynthesis (F).

The MetaboAnalyst 3.0 and KEGG online database were used to explore the potential disturbed metabolomic pathways that were related to GC. The pathway analysis results are described in Figure 6. The Y-axis (-log⁡(p)) was the log-transformation of the original P-value calculated from the enrichment analysis, and the X-axis represents the pathway impact value calculated from the pathway topology analysis [25]. In our study, pathways with an original P-value lower than 0.05 and an impact higher than 0.15 were selected as the significantly perturbed pathways. Finally, histidine metabolism (L-histidine, 1-methylhistidine, and L-glutamic acid), phenylalanine metabolism (L-phenylalanine and L-tyrosine), D-glutamine and D-glutamate metabolism (L-glutamic acid and L-glutamine), valine, leucine, isoleucine biosynthesis (L-leucine and L-isoleucine), and glycerophospholipid metabolism (LysoPC(18:1(9Z)), phosphorylcholine, acetylcholine, glycerol 3-phosphate, and glycerophosphocholine) were selected as the most perturbed metabolic pathways in the model group. Furthermore, to evaluate the metabolic alterations of mice treated with MSJZT and probe the underlying antitumour mechanism of the drug, the aforementioned metabolic pathways were taken as the detection indexes. We found three pathways were significantly altered in the MSJZT group as follows: histidine metabolism (L-histidine, 1-methylhistidine, urocanate), glycerophospholipid metabolism (LysoPC(18:1(9Z)), phosphorylcholine, glycerol 3-phosphate, glycerophosphocholine), D-glutamine, and D-glutamate metabolism (L-glutamic acid and L-glutamine).

**Figure 7:**
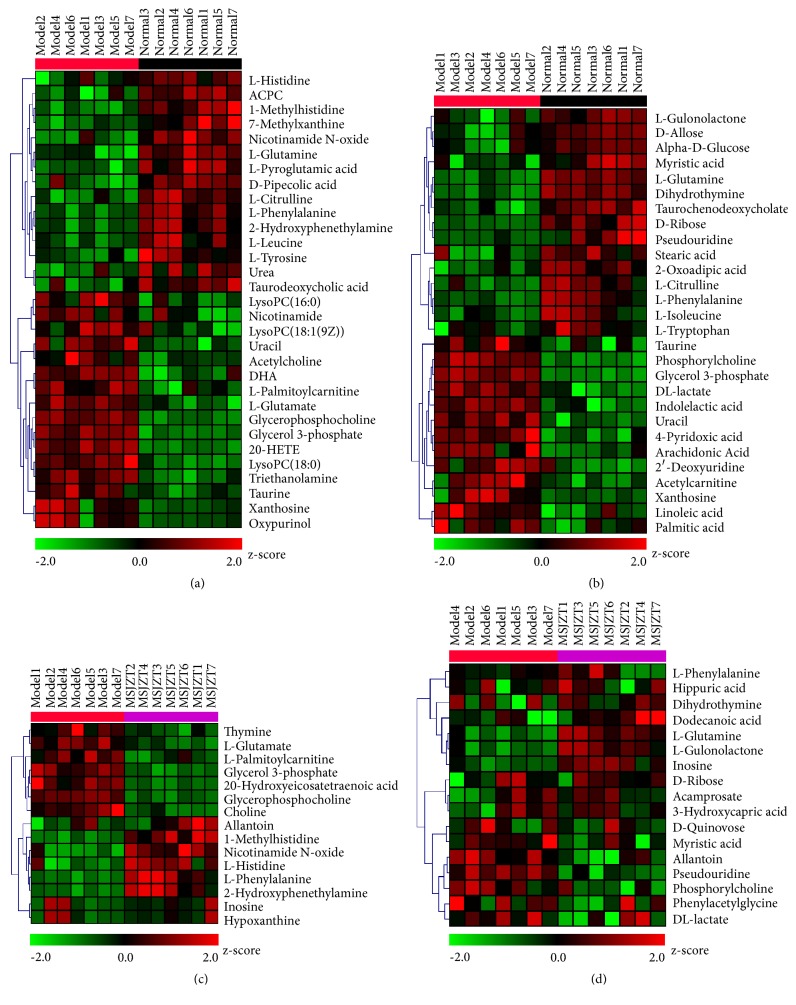
Heatmap visualization of differential metabolites. (a, b) The model* vs.* the normal group both in positive and negative modes. (c, d) The MSJZT* vs. *the model group both in positive and negative modes.

Significant metabolites were hierarchically clustered in a heatmap by Multi Experiment Viewer in Figure 7. Each column represents a sample of the groups, marked on the top, and each row corresponds to a “potential biomarker”. The colour indicates the metabolite expression value according to the different intensity level of the metabolites: red represents the highest level; and green represents the lowest. The heatmap indicates that, in the model group, most metabolites are shown in a red colour, and those in the normal group are displayed in green, while those in the MSJZT group were in between these two groups.

**Figure 8:**
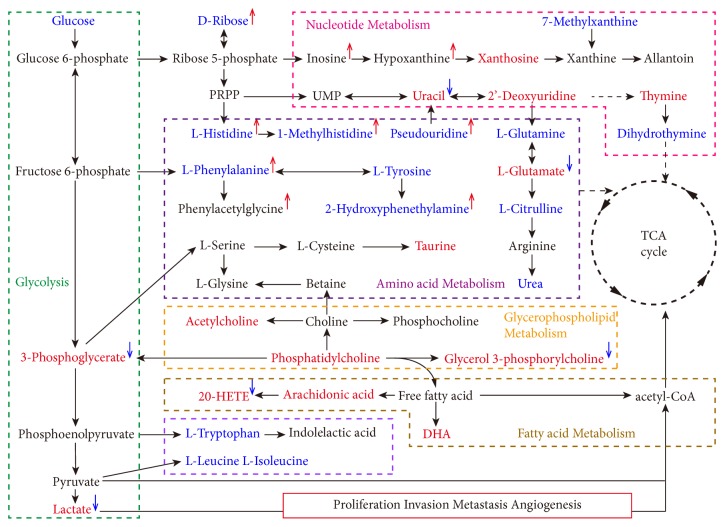
The integrative metabolism pathway map according to the KEGG pathway database. The perturbed metabolism pathways mainly includes glycolysis, nucleotide metabolism, amino acid metabolism, and lipid metabolism. The blue colour metabolites represent downregulated, while the red colour metabolites represent upregulated, in the model group. The blue arrows represent downregulated, and the red arrows represent upregulated, metabolites in the MSJZT group.

**Figure 9:**
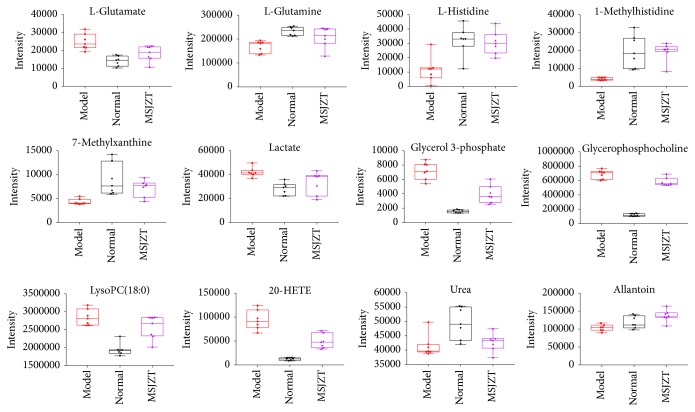
Blot plots of the relative intensity of related metabolites, which mainly included amino acids, glycolysis, lipid, and nucleotide metabolism.

Thus, we can conclude that MSJZT could reverse the biomarker trends. Based on the above results, a map that was related to GC and MSZJT was generated (Figure 8). Then, the primary related metabolites that played an important role in the disturbed pathways such as L-glutamate, L-glutamine, L-histidine, 1-methylhistidine, 7-methylxanthine, lactate, glycerol-3-phosphate, glycerophosphocholine, LysoPC(18:0), 20-HETE, urea, and allantoin are depicted in Figure 9.

### 3.8. Validation of LDH, GS, PCYT2 mRNA, and Protein Levels

**Figure 10:**
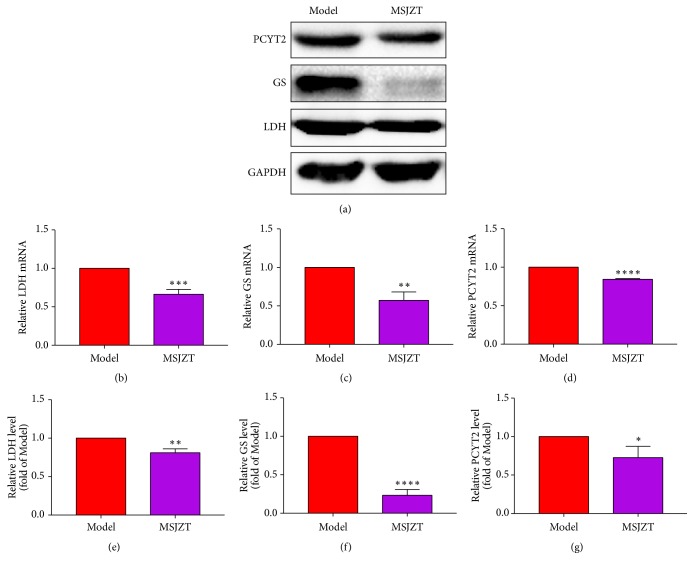
Relative expression of LDH, GS, and PCYT2 and their corresponding mRNA levels. (a) Image of western blot. (b)–(d) The relative mRNA of LDH, GS, and PCYT2, respectively. (e)–(g) The relative protein level of LDH, GS, and PCYT2, respectively. Data were presented as the mean ± SD (n=3). T-test between model group and MSJZT group, *∗*P<0.05, *∗∗*P<0.01, *∗∗∗*P<0.001, and *∗∗∗* *∗*P<0.0001. The experiment was repeated three times.

To validate the disordered pathways, we selected the important enzyme genes transcription levels of LDH, GS, and PCYT2 in model and MSJZT group to perform qRT-PCR to detect the mRNA. Western blotting analyses were used to detect the protein expression level of LDH, GS, and PCYT2. As shown in Figure 10, MSJZT remarkably reduce the expression of LDH, GS, and PCYT2 both in mRNA and protein level. These results revealed that MSJZT have the ability to rectify glycolytic, amino acid, and phospholipids metabolism in GC.

## 4. Discussion

### 4.1. Quality Control of MSJZT and the Metabolomic Method

In this study, MSJZT was prepared with strict quality control using a UPLC-PDA method. First, compared with the normal group, the body weight in the model group and 5-Fu group decreased, while the body weight in the MSJZT group reached 25.09 g. Therefore, MSJZT could effectively maintain the body weight of tumour-bearing mice and improve their appetite and, thus, may enhance their ability to resist tumour-induced cachexia. Throughout the whole experimental period, the tumour grew rapidly in the model group, and eventually the average tumour volume reached 952.69 mm^3^, while in the MSJZT group, the tumour volume reached 295.51 mm^3^. This finding indicated that MSJZT played an obvious role in blocking tumour growth. The histological assay showed that the GC model was successfully established, and MSJZT had an excellent antitumour activity.

The mouse plasma was used for the subsequent metabolomic analysis. Moreover, the use of quality control (QC) samples indicated that the analysis method is reliable and reproducible, and the data were of sufficient quality according to statistical analysis [26]. The two steps established a solid foundation for the metabolomics process. With further pathway analysis, our results revealed that MSJZT could repair the perturbed metabolic state of GC, which contributed to illustrating the pathogenesis of GC and the presumed mechanism of MSJZT.

### 4.2. Biological Explanation of the Metabolic Pathways Related to GC

GC is a main contributor to the global burden of disability-adjusted life-years from cancer and accounts for 20% of cancer cases worldwide [1]. The pathogenesis mainly includes* Helicobacter pylori* infection, hereditary susceptibility and environmental factors; however, the precise mechanisms remain unknown. Recently, the application of various “-omics” technologies has opened a new field to investigate the mechanisms behind this disease [27]. Metabolism in tumours plays a key role in cellular logistics for rapid proliferation, maintenance of redox homeostasis and in epigenetics and reprogramming [28]. In recent years, there have been many metabolomic studies on GC and reported various metabolites related to GC [29–31]. As a result, the perturbed pathways resulting from the metabolomic analysis of GC were similar, consistently showing a metabolism disorder of amino acids, lipids, and glycolysis in Figure 8. The perturbed pathways could be used to explore the association with the known pathogenesis mechanism of GC.

For cancer cells, their common feature lies in the ability to acquire necessary nutrients from a frequently nutrient-poor environment and utilizing these nutrients to both maintain viability and build new biomass. Glutamine and glucose are the two principal nutrients that support survival [32]. According to the data shown in Tables 4 and 5, the level of glutamine decreased in the model group, along with some free amino acids. Reprogramming of glutamine metabolism further contributes to the proliferative and metabolic responses regulated by the oncogenic transcription factor c-Myc [33]. Actually, the prominent role of glutamine in tumour-selective metabolic pathways has received considerable attention, which is evident from the widespread use of the terms such as “glutamine addiction” and “glutaminolysis” in the vocabulary of cancer biology [28, 34, 35]. It is not only an essential nitrogen donor for several key metabolic enzymes and the* de novo* synthesis of nucleic acids but also a precursor of alpha-ketoglutarate, which is a TCA intermediate, and continuation of this cycle generates additional energy to produce building blocks for cells [36]. The proposed mechanism is that c-Myc induces the transcription of glutamine transporters ASCT2, in addition, promotes the expression of the glutamine-utilizing enzymes glutaminase (GS), which support transporter-facilitated glutamine uptake by converting glutamine to glutamate [37–39]. Thus, glutamate cannot exit the cell through glutamine transport, and its accumulation further promotes TCA cycle anaplerosis, which makes tumour growth uncontrollable. Additionally, as a nonessential amino acid, it is an indispensable source of imido groups for the synthesis of purines and pyrimidines. Histidine, a precursor of histamine, is a vital inflammatory agent in immune responses; it displays antiapoptotic effects, acts as an antioxidant, and acts against excitotoxicity [40, 41].

Since the “Warburg effect” was first reported in 1956, by definition, cancer cells generally undergo glycolysis instead of oxidative phosphorylation for energy. Pyruvate generated from glycolysis is converted to lactate by lactic dehydrogenase (LDH) under hypoxic. The level of lactate, as the end-product of glycolysis, is increased in the model group, and the ability to internalize glucose is enhanced in GC, as shown in Table 4. Lactate accumulation creates a potentially favourable microenvironment for cancer cells to proliferate; it causes local acidosis and potentially modulates the activity of proteases that decompose the extracellular matrix, thereby liberating peptides and amino acids that are consumable for energy generation [42]. Furthermore, lactate accumulation is instrumental for the promotion of angiogenesis. Lactate promotes the stabilization of HIF-1*α* and activates NF-*κ*B and PI-3 kinase signalling in endothelial cells and induces the secretion of the proangiogenic factor VEGF from tumour-associated stromal cells. Increased levels of lactate also stimulate hyaluronic acid production by fibroblasts, which may contribute to tumour invasiveness [43]. With regard to glucose, PI3K/Akt signalling promotes both the expression of glucose transporter GLUT1 mRNA and the translocation of GLUT1 protein from the* endo *membranes to the cell surface. In addition, multiple growth signalling nodes that become aberrantly activated in cancer share an ability to facilitate cellular access to glucose, a principal metabolic substrate [32]. 3-phosphoglycerate, as an intermediate metabolite of glycolysis, is also increased. 3-phosphoglycerate can be further metabolized to form the glycerol component of triacylglycerides and membrane phospholipids and is a precursor for the biosynthesis of serine and glycine to generate methyl donor groups and NADPH [44].

A hallmark of malignant cancer cells is the enhanced flux of carbon into anabolic pathways to support rapid growth and proliferation at the expense of oxidative phosphorylation and ATP synthesis. It is known that cells possess a complex array of glycerolipids, including phospholipids, triacylglycerol (TAG), diacylglycerol (DAG), sphingolipids, and sterols, that are essential components of membranes and energy storage systems, cell signalling molecules, and effectors of protein structure and function [45]. In GC, many characteristics are amplified and modified. Choline is an essential nutrient that is synthesized* de novo* by the transfer of three methyl groups from S-adenosylmethionine to phosphatidylethanolamine to form phosphatidylcholine. Accelerated phosphatidylcholine synthesis that results from the overexpression of phosphocholine cytidylyltransferase (PCYT2) induces cells to undergo a growth factor-induced G0/G1 transition. Additionally, intrinsic apoptotic stimuli have been identified that target phosphatidylcholine metabolism by caspase-dependent mechanisms [45].

The rapid proliferation and differentiation of tumour cells also lead to significant dysfunction of nucleotide synthesis and catabolism. Nucleotides are associated with energy metabolism, mainly in the form of ATP and GTP [27]. Some nucleotide analogues, which can be used as anticancer drugs, can interfere with nucleotide metabolism. Accumulation of the end products of nucleotide catabolism is characterized by higher levels of uric acid or urate in GC patients and animal models. Other purine compounds, such as hypoxanthine and guanosine, are also increased [46, 47]. The level of urea and allantoin increased in the model group; they are the important metabolic end products of purine catabolism in the body in addition to being antioxidants. This finding revealed an elevated purine catabolism involved in the growth and proliferation of tumour cells [46]. 7-methylxanthine is a naturally occurring purine derivative and a reaction intermediate in the metabolism of adenosine and in the formation of nucleic acids [48]. The upregulated 7-methylxanthine indicated excess cell replication in GC.

### 4.3. Antitumour Effect of MSJZT

The main active components of MSJZT include atractylenolide III, ginsenoside Re, pachymic acid, glycyrrhizic acid, berberine, and oleanolic acid. Atractylenolide III has been reported to inhibit cell growth, increase lactate dehydrogenase release and modulate the cell cycle of human lung carcinoma A549 cells [49]. Ginsenoside Re can induce GC cells into a state of S arrest and activate Caspase-8, Caspase-9, and Caspase-3, followed by cleavage of PARP [50]. Pachymic acid can upregulate the expression of the proapoptotic protein Bax and downregulate the expression of the antiapoptotic protein Bcl-2, which are related to the function of mitochondria [51]. Glycyrrhizic acid induces human MDA-MB-231 breast cancer cell death and autophagy via the ROS-mitochondrial pathway [52]. Berberine reduces the growth of MGC 803 cells and IL-8 expression levels, which was associated with deactivation of the p38 MAPK, ERK1/2 and JNK signalling pathways [12]. Oleanolic acid induces apoptosis-independent autophagic cell death in multiple human GC cell lines via modulation of the expression of phospho-mTOR, which is associated with the inhibition of the PI3K/AKT and ERK/p38 MAPK signalling pathways and activation of the AMPK signalling pathway [53]. The results of our study indicated that MSJZT exhibited a good antitumour effect. It not only enhanced the body weight of the mice but it also shrank the tumour volume. Furthermore, the metabolomic results revealed that the perturbed pathways such as amino acid, glycolysis, and lipids metabolism were partly regulated by MSJZT through decreasing the content of LDH, GS, and PCYT2 mRNA and protein level. This finding provided sufficient evidence to support the concept that MSJZT has an integrated effect on the dysfunction of energy metabolism, which is probably related to cell cycle arrest, apoptosis, proliferation and inactivation of oncogenes.

On the other hand, the commonly used antineoplastic drugs, such as 5-Fu and Taxol, induce many inevitable complications and side effects, mainly including fatigue, pain, infection/fever, anaemia, diarrhoea, nausea and vomiting, hair loss, and bone marrow suppression [54]. In contrast, TCM formulae exhibit low toxicity, high efficiency, and multitargets and should be used as a priority in combination therapy in treating patients with cancer.

## 5. Conclusion

In this study, first, MSJZT was prepared following strict quality control with a UPLC-PDA method. Then, we established a subcutaneous transplantation tumour model of GC. After MSJZT administration, the mouse body weight increased, and the tumour growth inhibition rate reached 68.98% compared with the model group. Subsequently, a metabolomic method was used to explore the mechanism of action of MSJZT. The perturbed pathways, such as amino acid, glycolysis, lipids, and nucleotides metabolism, were partially regulated by MSJZT. These findings provide solid evidence that MSJZT has an integrative effect on the dysfunction of energy metabolism. These findings could not only establish the foundation for TCM to treat GC but also provide a basis for further exploration of the precise mechanism of MSJZT.

## Figures and Tables

**Table 1:** Components of modified Si Jun Zi Tang.

Chinese name	English name	Latin name	Ratio (g)	Batch number	Source province
Ren shen	Ginseng	*Panaxginseng *C.A.Mey.	10	17061608	Gansu
Bai zhu	Largehead Atractylodes	*Atractylis macrocephala *Desf.	10	17052501	Zhejiang
Fu ling	Indian Bread	*Poriacocos* (Schw.)Wolf	10	17070109	Yunnan
Gan cao	Ural Licorice	*Glycyrrhizauralensis *Fisch.	3	170701	Inner Mongolia
Huang lian	Chinese Goldthread	*Coptischinensis *Franch.	6	17032201	Sichuan
Bai hua she she cao	Oldenlandia diffusa	*Hedyotis diffusa* Willd.	20	17061609	Guangxi

**Table 2:** Specific primers for the qRT-PCR analysis.

Gene	Forward	Reverse
LDH	CATGGCCTGTGCCATCAGTA	GGAAAAGGCTGCCATGTTGG
GS	TCGATGGTACTGGAGAAGGACTGC	CATGGCAGCAGGCACGAGATAC
PCYT2	AGATGGTGCAGGCCATCAAA	TGCCGTGAACACAGAAGTCA
GAPDH	ACTAGGCGCTCACTGTTCTCT	AAATCCGTTGACTCCGACCT

**Table 3:** The detailed model parameters of the OPLS-DA and 200 permutation tests.

	Model - Normal		Model - MSJZT	
	positive	negative	positive	negative
R2X	0.347	0.442	0.264	0.352
R2Y	0.997	0.995	0.989	0.993
Q2	0.851	0.953	0.454	0.494
R2 intercept	0.948	0.845	0.969	0.984
Q2 intercept	-0.235	-0.342	-0.075	-0.035

**Table 4:** Differential metabolites among groups in positive ion mode.

Metabolites	m/z	RT(s)	HMDB	Normal *vs.* model		MSJZT *vs.* model			
				VIP	FC	P-value	VIP	FC	P-value
DHA	346.2739	68.843	HMDB0062579	6.248	0.660	<0.001	-	-	-
7-Methylxanthine	166.0523	74.755	HMDB0001991	1.102	2.064	0.004	-	-	-
20-HETE	338.2685	76.696	HMDB0005998	4.895	0.134	<0.001	3.945	0.530	0.001
Nicotinamide	123.0545	87.755	HMDB0001406	2.055	0.691	0.018	-	-	-
Uracil	113.0336	142.0695	HMDB0000300	-	-	-	2.409	0.541	<0.001
Thymine	127.0492	165.724	HMDB0000262	1.116	0.716	0.005	-	-	-
Urea	61.03878	172.386	HMDB0000294	1.673	1.194	0.008	-	-	-
Taurodeoxycholic acid	517.3293	238.846	HMDB0000896	1.234	3.026	0.013	-	-	-
Triethanolamine	150.1116	269.222	HMDB0032538	1.030	0.296	<0.001	-	-	-
Nicotinamide N-oxide	139.0494	273.546	HMDB0002730	2.009	2.168	<0.001	1.840	2.317	<0.001
L-Palmitoylcarnitine	400.3417	293.014	HMDB0000222	2.708	0.599	0.001	2.128	0.733	0.017
Allantoin	176.0772	328.55	HMDB0000462	-	-	-	1.092	1.175	0.003
LysoPC(18:0)	524.3704	336.747	HMDB0010384	14.905	0.682	<0.001	3.465	0.857	0.032
LysoPC(18:1(9Z))	522.3538	339.1875	HMDB0002815	3.272	0.863	0.022	-	-	-
LysoPC(16:0)	496.339	382.673	HMDB0010382	2.782	0.722	0.008	-	-	-
Inosine	269.0878	396.6175	HMDB0000195	-	-	-	5.884	6.061	0.034
Hypoxanthine	137.045	396.832	HMDB0000157	-	-	-	9.224	7.951	0.039
2-Hydroxyphenethylamine	120.0798	469.973	HMDB0001065	2.841	1.594	0.002	1.237	1.350	0.033
L-Phenylalanine	166.0857	469.984	HMDB0000159	3.928	1.656	0.001	1.963	1.380	0.026
L-Leucine	132.1011	483.5645	HMDB0000687	4.807	1.871	0.008	-	-	-
Taurine	126.0214	549.757	HMDB0000251	2.156	0.774	0.007	-	-	-
L-Tyrosine	182.0804	555.5655	HMDB0000158	1.041	1.509	0.008	-	-	-
D-Pipecolic acid	130.0853	573.452	HMDB0005960	2.262	2.146	0.004	-	-	-
Oxypurinol	153.04	595.101	HMDB0000786	1.085	0.079	0.031	-	-	-
Xanthosine	285.0826	595.101	HMDB0000299	1.597	0.076	0.031	-	-	-
L-Glutamine	147.076	701.805	HMDB0000641	4.637	1.378	<0.001	-	-	-
L-Pyroglutamic acid	130.0493	701.8085	HMDB0000267	3.973	1.249	<0.001	-	-	-
ACPC	84.04351	702.062	HMDB0036458	1.258	1.322	<0.001	-	-	-
Acetylcholine	146.117	706.122	HMDB0000895	1.773	0.696	0.027	-	-	-
1-Methylhistidine	170.0919	722.94	HMDB0000001	2.122	4.723	0.001	2.043	4.670	<0.001
Glycerophosphocholine	258.1097	729.043	HMDB0000086	8.256	0.619	<0.001	8.243	0.847	0.006
L-Citrulline	176.1023	736.684	HMDB0000904	1.982	1.356	0.001	-	-	-
L-Glutamate	148.0598	761.895	HMDB0000148	1.559	0.564	<0.001	1.872	0.740	0.018
L-Histidine	156.076	761.9205	HMDB0000177	2.418	2.725	0.002	1.436	2.612	0.001
Glycerol 3-phosphate	173.0203	835.814	HMDB0000126	1.290	0.212	<0.001	1.238	0.551	<0.001

Notes: metabolites: potential biomarkers; RT: retention time; VIP: variable importance in the projection; FC: fold change; 20 HETE: 20-hydroxyeicosatetraenoic acid; ACPC: 1-aminocyclopropanecarboxylic acid.

**Table 5:** Differential metabolites among groups in negative ion mode.

Metabolites	m/z	RT(s)	HMDB	Normal *vs. *Model			MSJZT *vs.* model		
				VIP	FC	P-value	VIP	FC	P-value
Acamprosate	180.0331	61.982	HMDB0014797	-	-	-	1.161	0.654	0.009
Acetylcarnitine	405.227	65.483	HMDB0000201	2.415	0.335	0.003	-	-	-
4-Pyridoxic acid	182.0455	67.1	HMDB0000017	1.601	0.478	<0.001	1.167	1.360	0.004
Arachidonic Acid	303.2328	68.26	HMDB0060102	6.673	0.861	0.005	-	-	-
Stearic acid	567.5289	70.053	HMDB0000827	1.329	1.677	0.024	-	-	-
Linoleic acid	279.233	71.276	HMDB0000673	16.236	0.816	0.002	-	-	-
Palmitic acid	511.4722	72.976	HMDB0000220	2.605	0.885	0.048	-	-	-
Myristic acid	227.2015	74.892	HMDB0000806	7.495	1.401	0.001	8.339	1.240	0.014
Dodecanoic acid	199.1701	76.7325	HMDB0000638	-	-	-	2.971	1.169	0.032
3-Hydroxycapric acid	187.1335	78.2555	HMDB0002203	-	-	-	1.522	1.361	0.023
Uracil	111.0198	143.866	HMDB0000300	5.091	0.507	<0.001	-	-	-
L-Gulonic gamma-lactone	177.0403	189.902	HMDB0003466	5.649	2.119	0.001	-	-	-
2′-Deoxyuridine	227.0674	198.477	HMDB0000012	1.969	0.736	0.008	-	-	-
Taurochenodeoxycholate	498.2884	222.406	HMDB0000951	2.638	5.607	<0.001	-	-	-
Indolelactic acid	204.0664	257.5135	HMDB0000671	1.019	0.502	<0.001	-	-	-
Allantoin	157.0364	329.079	HMDB0000462	-	-	-	3.841	1.332	<0.001
Phenylacetylglycine	192.0665	365.929	HMDB0000821	-	-	-	1.494	1.877	0.007
Hippuric acid	178.0507	375.18	HMDB0000714	-	-	-	1.181	0.541	0.018
Inosine	267.0734	399.819	HMDB0000195	-	-	-	6.101	11.093	0.034
D-Quinovose	223.0822	423.6645	NA	-	-	-	1.141	3.170	0.010
DL-lactate	89.02428	445.111	HMDB0000755	10.283	0.699	<0.001	1.765	0.787	0.037
Pseudouridine	243.0619	451.015	HMDB0000767	1.293	1.381	0.006	2.082	1.419	0.002
L-Tryptophan	203.0826	470.464	HMDB0000929	2.132	1.405	0.028	-	-	-
L-Phenylalanine	164.0716	471.676	HMDB0000159	3.089	1.835	0.002	3.308	1.439	0.016
L-Isoleucine	130.0873	485.879	HMDB0000172	4.704	2.277	0.015	-	-	-
Taurine	124.0072	553.905	HMDB0000251	1.629	0.798	0.026	-	-	-
D-Allose	239.0772	563.703	HMDB0001151	4.919	2.221	<0.001	-	-	-
Alpha-D-Glucose	179.0563	564.292	HMDB0003345	6.480	1.791	0.001	-	-	-
D-Ribose	149.0452	579.172	HMDB0000283	1.404	2.149	<0.001	1.246	2.388	0.016
Xanthosine	283.0684	597.003	HMDB0000299	1.830	0.081	0.030	-	-	-
2-Oxoadipic acid	141.0169	628.85	HMDB0000225	7.548	1.062	0.001	-	-	-
L-Glutamine	145.0619	703.715	HMDB0000641	4.210	1.699	<0.001	4.608	1.404	0.002
Dihydrothymine	127.0509	703.732	HMDB0000079	1.113	1.667	<0.001	-	-	-
Phosphorylcholine	242.0798	731.901	HMDB0001565	1.185	0.584	<0.001	1.163	0.803	0.005
L-Citrulline	174.0884	738.368	HMDB0000904	1.288	1.536	0.002	-	-	-
Glycerol 3-phosphate	171.0062	836.0675	HMDB0000126	1.183	0.620	0.003	1.253	0.214	<0.001

Notes: metabolites: potential biomarkers; RT: retention time; VIP: variable importance in the projection; FC: fold change; P-value: calculated by Student's t-tests.

## Data Availability

The data used to support the findings of this study are available from the corresponding author upon request.

## Abbreviations

MSJZT: Modified Si Jun Zi Tang
GC: Gastric cancer
5-Fu: 5-Fluorouracil
HILIC UPLC-Q-TOF/MS: Hydrophilic interaction liquid chromatography and liquid chromatography quadrupole time-of-flight mass spectrometry
UPLC-PDA: Ultra performance liquid chromatography and photodiode array detector
QC: Quality control
PCA: Principal component analysis
OPLS-DA: Orthogonal partial least squares analysis
LDH: Lactic dehydrogenase
GS: Glutamine synthetase
PCYT2: Phosphocholine cytidylyltransferase
ASCT2: Glutamine transporter.

## Supplementary Materials

Supplementary Materials 
*Figure S1*: representative chromatogram of MSJZT and reference standards;* Figure S2*: the representative chromatogram of the reference standards.* Table S1*: the regression equation of 6 reference standards.* Table S2*: the content of 6 herbs of MSJZT (mg/g);* Table S3*: the body weight at third weeks (g);* Table S4*: the volume of tumour (mm^3^).
