# Supplementary material for: Metabolomic Study on Nude Mice Models of Gastric Cancer Treated with Modified Si Jun Zi Tang via HILIC UHPLC-Q-TOF/MS Analysis
Source: Evid Based Complement Alternat Med. 2019 Jun 23;2019:3817879. doi: 10.1155/2019/3817879 (PMC6612382; doi:10.1155/2019/3817879)
Supplement: Supplementary Materials — Figure S1: representative chromatogram of MSJZT and reference standards; Figure S2: the representative chromatogram of the reference standards. Table S1: the regression equation of 6 reference standards. Table S2: the content of 6 herbs of MSJZT (mg/g); Table S3: the body weight at third weeks (g); Table S4: the volume of tumour (mm3). [file 3817879.f1.docx]

**Appendix A. Supplementary material**

**Metabolomic Study on Nude Mice models of Gastric Cancer treated with Modified Si Jun Zi Tang via HILIC UHPLC-Q-TOF/ MS Analysis**

Shanshan Nie, Yuhang Zhao, Wenbo Wang, Ye Yao, Min Yi, Dongsheng Wang*

A

6

5

4

3

2

1

4

B

2

1

3

6

5

**Fig.S1** Representative chromatogram of A (Modified Si Jun Zi Tang) and B (Reference Standards). 1: Glycyrrhizic Acid; 2: Berberine; 3: Ginsenoside Re; 4: Atractylenolide III; 5: Pachymic acid; 6: Oleanolic acid;

A

Ginsenoside Re

B

Glycyrrhizic Acid

C

Pachymic acid

D

Oleanolic acid

E

Atractylenolide III

F

Berberine

**Fig.S2** The representative chromatogram of Reference Standards.

**TableS1** The regression equation of 6 reference standards.

| Analyte | regression equation | R2 |
| --- | --- | --- |
| Ginsenoside Re | Y=17065x-850.18 | 0.9999 |
| Pachymic acid | Y=60655x-3243.1 | 0.9999 |
| Oleanolic acid | Y=26759x+8402.4 | 0.9995 |
| Atractylenolide III | Y=58328x-8727 | 0.9998 |
| Glycyrrhizic acid | Y=33243x+22217 | 0.9994 |
| Berberine | Y=193085x-48217 | 0.9997 |

**TableS2** The content of 6 herbs of Modified Si Jun Zi Tang (mg/g)

| Analyte | Sample1 | Sample2 | Sample3 | mean | SD | RSD(%) |
| --- | --- | --- | --- | --- | --- | --- |
| Ginsenoside Re | 4.46 | 4.48 | 4.49 | 4.48 | 0.02 | 0.34 |
| Pachymic acid | 0.96 | 0.97 | 0.97 | 0.97 | 0.01 | 0.6 |
| Oleanolic acid | 0.97 | 1.01 | 0.98 | 0.99 | 0.02 | 2.11 |
| Atractylenolide III | 0.56 | 0.55 | 0.55 | 0.55 | 0.01 | 1.04 |
| Glycyrrhizic acid | 1.62 | 1.63 | 1.64 | 1.63 | 0.01 | 0.61 |
| Berberine | 1.53 | 1.54 | 1.51 | 1.53 | 0.02 | 1 |

**TableS3** The body weight at 21days (mean ± SD)

| Group | Mean ± SD | P value |
| --- | --- | --- |
| Model | 23.29 ± 0.85 | - |
| 5-Fu | 23.59 ± 0.88 | 0.0025^##^ |
| MSJZT | 25.09 ± 0.57 | 0.0055*** |
| Normal | 25.71 ± 0.83 | 0.0001**** |

Note: the model vs other groups, symbols****, ***mean differences were highly significant, *P*<0.001, very significant, *P*<0.01, respectively; the 5-Fu vs MSJZT group, ## represent *P*<0.01.

**TableS4** The volume of tumor(mm^3^) (mean ± SD)

| Group | Mean ± SD | inhibition rate (%) |
| --- | --- | --- |
| Model | 952.69 ± 115.75 | - |
| MSJZT | 295.51 ± 49.74 | 68.98± 0.05 |
| 5-Fu | 354.81 ±59.53 | 62.76 ±0.06 |
